# Prevalence and Antimicrobial Resistance of *Staphylococcus aureus* and *Staphylococcus schleiferi* Isolated from Dogs with Otitis Externa and Healthy Dogs

**DOI:** 10.3390/antibiotics14121194

**Published:** 2025-11-24

**Authors:** Ionela Popa, Ionica Iancu, Vlad Iorgoni, Janos Degi, Alexandru Gligor, Kalman Imre, Emil Tîrziu, Timea Bochiș, Călin Pop, Ana-Maria Plotuna, Paula Nistor, Marius Pentea, Viorel Herman, Ileana Nichita

**Affiliations:** 1 Department of Semiology, Faculty of Veterinary Medicine, University of Life Sciences “King Mihai I”, 300645 Timişoara, Romania; ionela.popa@usvt.ro (I.P.); timea.bochis@usvt.ro (T.B.); calinpop@usvt.ro (C.P.); 2 Department of Infectious Diseases and Preventive Medicine, Faculty of Veterinary Medicine, University of Life Sciences “King Mihai I”, 300645 Timişoara, Romania; vlad.iorgoni@usvt.ro (V.I.); janosdegi@usvt.ro (J.D.); alexandru.gligor@usvt.ro (A.G.); paula.nistor@usvt.ro (P.N.); viorel.herman@fmvt.ro (V.H.); 3 Department of Food Safety and Hygiene, Faculty of Veterinary Medicine, University of Life Sciences “King Mihai I”, 300645 Timişoara, Romania; kalmanimre@usvt.ro; 4 Department of Microbiology, Faculty of Veterinary Medicine, University of Life Sciences “King Mihai I”, 300645 Timisoara, Romania; emiltarziu@yahoo.com (E.T.); ileananichita@usvt.ro (I.N.); 5 Department of Animal Nutrition, University of Life Sciences “King Mihai I”, 300645 Timisoara, Romania; anamaria.plotuna@usvt.ro; 6 Department of Anatomy, University of Life Sciences “King Mihai I”, 300645 Timisoara, Romania; mariuspentea@usvt.ro

**Keywords:** *Staphylococcus aureus*, *Staphylococcus schleiferi*, canine otitis, MALDI-TOF MS, AMR surveillance, companion animals

## Abstract

**Background/Objectives**: Antimicrobial resistance (AMR) in companion animals is a growing One Health concern due to the close interaction between pets and humans. *Staphylococcus aureus* (*S. aureus*) and *Staphylococcus schleiferi* (*S. schleiferi*) are common colonizers of the canine ear canal and can act as reservoirs of resistance. This study aimed to assess the prevalence and antimicrobial resistance profiles of *S. aureus* and *S. schleiferi* isolated from dogs with otitis externa and clinically healthy dogs in western Romania. **Methods**: A total of 973 canine ear swabs were collected, 503 from dogs with otitis externa and 470 from healthy dogs. Isolates were identified using MALDI-TOF MS. Antimicrobial susceptibility testing was performed using the VITEK^®^ 2 Compact system, bioMérieux, Marcy-l’Étoile, France, and interpreted according to CLSI VET01 guidelines, with 13 antimicrobials representing multiple drug classes. **Results**: *S. aureus* was more prevalent in healthy dogs (20%) than in otitis cases (4%), while *S. schleiferi* was more common in otitic samples (7.5% vs. 4%). Among *S. aureus* isolates from otitic dogs, penicillin resistance was highest (65%), and 25% were multidrug-resistant (MDR). In healthy dogs, *S. aureus* showed 54.3% penicillin resistance and 16% MDR prevalence. Four MRSA strains (4.3%) were identified only in healthy dogs. *S. schleiferi* exhibited the highest resistance to clindamycin, with MDR rates of 10.6% in otitic and 5.6% in healthy dogs. No MRSS strains were detected. **Conclusions**: Clinically healthy dogs may serve as asymptomatic carriers of resistant *Staphylococcus* strains, including MRSA. Routine antimicrobial susceptibility testing is essential to inform treatment choices and mitigate resistance dissemination within veterinary and public health contexts.

## 1. Introduction

Antimicrobial resistance (AMR) is an increasing global health threat with significant consequences for healthcare systems worldwide [1,2,3,4,5,6].

The rapid emergence and dissemination of multidrug-resistant (MDR) bacterial pathogens represent growing concerns for public health worldwide. The World Health Organization has consistently identified AMR as one of the most critical threats to global health [7]. In recent years, there has been a marked increase in the number of clinical cases associated with MDR organisms, affecting both human and veterinary medicine. This concerning trend is compounded by the steady decline in the efficacy of available antimicrobial therapies, contributing to heightened morbidity and mortality rates [1,7,8,9].

Notably, AMR is now widely recognized as a One Health challenge reflecting the complex interconnection between human, animal, and environmental health [8,10,11,12,13]. MDR bacteria are typically defined as those exhibiting resistance to antimicrobial agents across no fewer than three antimicrobial classes [14].

Dogs and cats, as common household pets, have increasingly been regarded as possible carriers of AMR, primarily due to their frequent exposure to broad-spectrum antimicrobials and their close proximity to humans [1,3,15,16]. Moreover, intensified human-animal interactions not only contribute to the spread of drug-resistant bacteria but also facilitate the emergence of zoonotic infections [15,17,18,19,20,21]. Should multidrug-resistant organisms be present among domestic pets, the chances of treatment failure may significantly rise for both humans and animals. Therefore, gaining insight into the occurrence of AMR in companion species, particularly dogs and cats, is a growing priority in both veterinary and human health sectors. However, due to limited and inconsistent clinical reporting, the current body of data concerning AMR in pets remains insufficient [3].

A clinical report described an elderly patient with chronic illnesses who developed a methicillin-resistant *S. aureus* (MRSA) infection during hospitalization. Shortly afterward, his dog exhibited skin lesions and was also found to be infected with MRSA. The timing and clinical context suggest that the bacteria were transmitted between the owner and his pet, most likely from the human to the dog [22].

MRSA is recognized as a major global health concern [7,23,24,25,26,27,28,29]. It is thought that between 20% and 30% of individuals may carry *S. aureus* persistently, with the bacterium commonly residing on the skin and inside the nasal passages as part of the normal microbial flora [16,21,24]. Moreover, the cutaneous and ear canal microbial communities found in healthy pet dogs encompass diverse *Staphylococcus* species, among them the clinically significant MRSA [30].

In addition, the increased isolation of *S. schleiferi* from dogs without evident clinical manifestations, as well as from dogs presenting conditions such as otitis externa or pyoderma, represents a growing concern in contemporary veterinary medicine. This is primarily attributed to its marked antimicrobial resistance and its diverse repertoire of virulence determinants [31].

A case report describes a potential instance of zoonotic transmission involving *S. schleiferi* subsp. *coagulans* bloodstream infection, which occurred subsequent to a canine episode of otitis externa culminating in severe hemodynamic compromise and vascular infection, ultimately requiring intensive care management and vasopressor therapy in an otherwise immunocompetent patient [32].

Otitis externa in dogs is a frequently encountered condition marked by a diverse and multifactorial etiology [33].

Thorough profiling of the indigenous staphylococcal microbial community within the canine external auditory canal is vital for understanding both disease causation and transmission dynamics in staphylococcal otitis externa [33].

Evaluating antimicrobial susceptibility in bacterial isolates belonging to the genus Staphylococcus is critical for informing rational chemotherapeutic strategies, assessing the activity of novel antimicrobial compounds, and tracking resistance development due to prolonged exposure of field strains [33].

The aim of this study was to evaluate the prevalence and antimicrobial resistance profiles of *S. aureus* and *S. schleiferi* strains isolated from ear secretions of dogs with otitis externa, compared to those obtained from clinically healthy dogs in western Romania. Additionally, the study sought to identify MDR strains, as well as MRSA and methicillin-resistant *S. schleiferi* (MRSS) strains, to highlight the potential role of healthy dogs as reservoirs of antimicrobial resistance in the community.

## 2. Results

### 2.1. Prevalence of S. aureus and S. schleiferi in Dogs with Otitis Externa and Healthy Dogs

*S. aureus* was isolated in 4% of samples (*n* = 20) collected from dogs diagnosed with otitis externa. In contrast, the prevalence of *S. aureus* in samples obtained from the ears of clinically healthy dogs was notably higher, at 20% (*n* = 94). Regarding *S. schleiferi*, this species was isolated in 7.5% of samples (*n* = 38) from dogs with otitis externa, whereas its prevalence in samples from healthy canine ears was 4% (*n* = 18).

### 2.2. Antimicrobial Resistance Profiles of S. aureus and S. schleiferi from Dogs with Otitis Externa and Healthy Dogs

**Figure 1:**
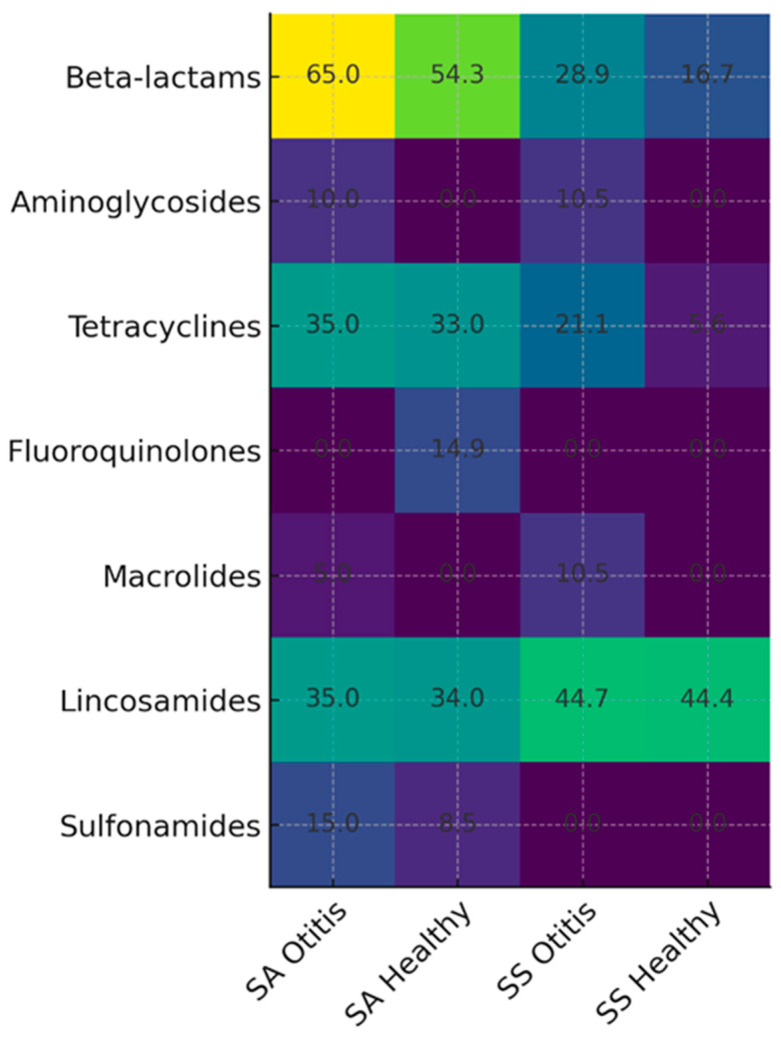
Heatmap illustrating the antimicrobial resistance percentages of *S. aureus* and *S. schleiferi* isolated from dogs with otitis externa and clinically healthy dogs. The color scale represents resistance levels (%) across major antimicrobial classes, allowing direct comparison between the four groups (SA–Otitis, SA–Healthy, SS–Otitis, SS–Healthy). Increased resistance to β-lactams in *S. aureus* and to lincosamides in *S. schleiferi* is highlighted as predominant resistance trends.

Regarding AMR, *S. aureus* strains isolated from dogs with otitis externa exhibited the highest resistance to penicillin, with 65% of isolates (*n* = 13) being resistant. In contrast, all isolates (100%, *n* = 20) were fully susceptible to oxacillin, ciprofloxacin, moxifloxacin, linezolid, teicoplanin, vancomycin, fusidic acid, and tigecycline (Table 1). As for *S. schleiferi* isolates from dogs with otitis externa, the highest resistance rate was observed against clindamycin, at 44.7% (*n* = 17). However, all isolates (100%, *n* = 38) demonstrated full susceptibility to oxacillin, ciprofloxacin, moxifloxacin, linezolid, teicoplanin, vancomycin, fusidic acid, tigecycline, and the combination of trimethoprim-sulfamethoxazole (Table 1, Figure 1).

Regarding AMR in strains isolated from the ears of healthy dogs, *S. aureus* strains exhibited the highest resistance to penicillin at 54.3% (*n* = 51). Conversely, no resistance was observed against gentamicin, moxifloxacin, erythromycin, linezolid, teicoplanin, and tigecycline, with all isolates (100%, *n* = 94) demonstrating susceptibility (Table 1, Figure 1).

In the case of *S. schleiferi* strains, the highest resistance was observed against clindamycin, with a prevalence of 44.4% (*n* = 8). However, no resistance was detected against oxacillin, gentamicin, ciprofloxacin, moxifloxacin, erythromycin, linezolid, teicoplanin, vancomycin, fusidic acid, tigecycline, and trimethoprim-sulfamethoxazole, with full susceptibility reported in 100% of isolates (*n* = 18) (Table 1, Figure 1).

### 2.3. Antimicrobial Resistance by Antimicrobial Class

Both *S. aureus* strains isolated from dogs with otitis externa and those isolated from the ears of healthy dogs exhibited the highest resistance to antibiotics belonging to the β-lactam class (Table 2). Similarly, *S. schleiferi* strains, regardless of whether they were isolated from dogs with otitis externa or from healthy ears, showed the highest resistance to antibiotics from the lincosamide class (Table 2).

### 2.4. MDR Strains

Regarding MDR strains, among *S. aureus* isolates from dogs with otitis externa, 5 strains were identified as MDR (25%). Similarly, among *S. aureus* strains isolated from the ears of healthy dogs, 15 strains were MDR (16%) (Table 3 and Table 4).

In the case of *S. schleiferi*, among strains isolated from dogs with otitis externa, 4 strains were MDR (10.6%), while among strains isolated from the ears of healthy dogs, one strain was MDR (5.6%) (Table 5 and Table 6).

Regarding MRSA strains, these were isolated exclusively from healthy dogs, with a prevalence of 4.3% (*n* = 4) (Table 4), and were not detected in dogs with otitis externa (Table 3). In addition to resistance to β-lactam antibiotics, these strains also exhibited resistance to from antimicrobials other classes (Table 4).

As for MRSS strains, they were not isolated from any of the samples collected from dogs with otitis externa or from healthy dogs (Table 5 and Table 6).

## 3. Discussion

In this study, the prevalence of *S. aureus* isolated from dogs with otitis externa is 4%, our results being similar to those obtained in the study conducted by Lee et al. [31], in which the prevalence of *S. aureus* isolated from dogs with otitis externa was 3%. Unlike the results obtained in this study, the results obtained in some studies showed a higher prevalence; specifically, the results obtained in the study conducted by Hassan et al. [34] showed a prevalence of 22.2%, and the results obtained in the study conducted by Öztürk et al. [35] showed a prevalence of 23.6%. Additionally, similar findings were reported in the study conducted by Chehida et al. [36], where the prevalence of *S. aureus* was 20.2%, isolated from different parts of the dogs’ bodies. These differences may be attributed to factors such as geographical diversity or bacterial isolation methods.

Regarding the penicillin resistance of *S. aureus* strains isolated from dogs with otitis externa, in the present study, it was 65% (Table 1); our results are2 similar to those reported by Bourély et al. [37], where *S. aureus* exhibited penicillin resistance at a rate of 70.9%. These findings can be interpreted as indicators of a persistent circulation of resistant strains within the veterinary environment. Concerning gentamicin resistance, in the present study, it was 10% (Table 1), a value comparable to that reported by Bourély et al. [37] (12.9%). However, the results obtained by Penna et al. [38] indicate a significantly higher resistance rate of 84.6%. These differences may reflect variations in antimicrobial usage across the respective regions.

Erythromycin resistance observed in this study among *S. aureus* strains was 5% (Table 1), differing from the results reported by Bourély et al. [37] (30.2%) and Penna et al. [38] (84.6%). Regarding clindamycin resistance, in the present study, *S. aureus* exhibited a resistance rate of 35% (Table 1), which differs from the findings reported by Saputra et al. [39], where *S. aureus* isolated from various infections showed clindamycin resistance of 2.1%. For tetracycline, the resistance observed in this study was 35% (Table 1), whereas Saputra et al. [39] reported a lower value of 10.6%. Concerning ciprofloxacin, *S. aureus* isolated from dogs with otitis externa showed no resistance (Table 1), which differs from the study by Saputra et al. [39], reporting a resistance of 8.5%, and from Penna et al. [38], who indicated a rate of 53.8%. This finding suggests a potential conserved efficacy of fluoroquinolones in certain regions and underscores the importance of continuous surveillance of resistance profiles to prevent the loss of this valuable therapeutic agent. Resistance to trimethoprim-sulfamethoxazole in this study was 15% (Table 1), a value close to that reported by Bourély et al. [37] (10.2%). Saputra et al. [39] demonstrated a slightly lower resistance rate of 6.4%.

In the present study, *S. aureus* was isolated from the ears of healthy dogs at a prevalence of 20%, unlike the findings of Lee et al. [31], who did not identify *S. aureus* in healthy dogs.

In our study, *S. aureus* isolated from the ears of healthy dogs exhibited resistance to ciprofloxacin at a rate of 14.9% (Table 1), which is lower than the 40.7% resistance reported by Wedley et al. [40] in *S. aureus* strains isolated from canine nasal samples. Regarding resistance to fusidic acid, the rate observed in this study was 2.1% (Table 1), markedly lower than the 53.7% resistance reported by Wedley et al. [40] for nasal isolates. Additionally, *S. aureus* strains isolated from healthy dogs’ ears demonstrated a tetracycline resistance rate of 33% (Table 1), substantially higher than the 1.9% reported by Wedley et al. [40]. Concerning vancomycin resistance, the strains in our study showed low resistance at 2.1% (Table 1), which aligns with the findings of Wedley et al. [40], where no vancomycin resistance was detected in nasal isolates of *S. aureus* from dogs.

Regarding the prevalence of MRSA strains isolated from the ears of healthy dogs, in the present study, it was 4.3%, a finding comparable to that reported by Katakweba et al. [41] in Morogoro, Tanzania, where the prevalence of MRSA strains isolated from the oral cavity and perineal region of healthy dogs was 3%. In the study conducted by Khermouche et al. [42] in Algeria, MRSA was isolated in 6.8% of samples collected from different body sites of dogs, a prevalence relatively close to that observed in the present study. Our results differ from those obtained by Loeffler et al. [43], who reported a MRSA prevalence of 0.66% in isolates from the oral cavity, nostrils, perianal area, and axilla of dogs.

It should be noted that, in the present study, no subspecies-level differentiation was performed between *S. schleiferi* subsp. *schleiferi* and subsp. *coagulans* (*S. schleiferi* subsp. *coagulans* has since been reclassified as a distinct species, now referred to as *Staphylococcus coagulans*) [44]. Consequently, the results obtained were compared both with studies that do not make this distinction and with those analyzing the two subspecies separately, depending on the available information in the literature.

Regarding the prevalence of *S. schleiferi* isolated from dogs with otitis externa, the present study reports a rate of 7.5%, a value close to that reported in the study conducted by Dégi et al. [45], in which the prevalence of *S. schleiferi* isolated from dogs with otitis externa was 9.21%.

The resistance to penicillin among *S. schleiferi* strains isolated from cases of otitis externa was 28.9% in the present study (Table 1), a slightly lower value compared to that reported in the study conducted by Dégi et al. [45], in which a resistance rate of 42.85% was recorded. This difference could be explained by variations in the number of strains analyzed.

Regarding clindamycin resistance in *S. schleiferi* isolated from cases of otitis externa, the present study, reports a rate of 44.7% (Table 1), which is significantly higher than that reported by Rosales et al. [46] (6.3%), yet lower than the rate observed in the study by Palomino-Farfán et al. [47] (70.6%). For ciprofloxacin, *S. schleiferi* isolated from cases of otitis externa in the present study showed no resistance (Table 1), in contrast to the findings of Palomino-Farfán et al. [47] (58.8%) and Penna et al. [38], who reported resistance rates of 25% for subsp. *coagulans* and 57.1% for subsp. *schleiferi.* In our study, *S. schleiferi* exhibited resistance to erythromycin in 10.5% of cases (Table 1), results that differ from those reported by Penna et al. [38], where *S. schleiferi* subsp. *coagulans* showed resistance in 75% of isolates and *S. schleiferi* subsp. *schleiferi* in 92.8%. Regarding gentamicin, *S. schleiferi* isolated from otitis externa demonstrated a resistance rate of 10.5% (Table 1), which was markedly lower compared to the data reported by Penna et al. [38]: 62.5% for subsp. *Coagulans* and 85.7% for subsp. *Schleiferi.*

The unexpected observation that MRSA isolates were detected exclusively in clinically healthy dogs may reflect ecological dynamics related more to colonization than to active infection. Asymptomatic nasal and skin carriage of MRSA in companion animals is well documented and may occur independently of clinical disease. In addition, healthy dogs may differ in their past exposure to antimicrobial treatments compared to dogs with otitis externa, which could influence selective pressures and favor silent colonization rather than overt infection. Human–pet contact patterns may also contribute to intermittent MRSA acquisition without clinical manifestation, further supporting the hypothesis that these findings represent colonization events rather than disease-associated strains.

In the present study, *S. schleiferi* was isolated from the ears of healthy dogs in 4% of cases, a prevalence similar to that reported by Lee et al. [31], who identified *S. schleiferi* in 5% of healthy dogs.

Comparatively, *S. aureus* was more frequently isolated from the ears of healthy dogs (20%) than from those with otitis externa (4%). However, isolates from otitic ears showed a higher frequency of MDR strains (25% vs. 16%) and a greater resistance rate to penicillin (65% vs. 54.3%). Additionally, MRSA strains were detected only in healthy dogs (4.3%). In the case of *S. schleiferi,* the prevalence was higher in dogs with otitis (7.5%) compared to healthy individuals (4%). The highest resistance in both groups was observed against clindamycin (44.7% in dogs with otitis vs. 44.4% in healthy dogs). MDR strains were also more frequently identified in otitic samples (10.6% vs. 5.6%). No MRSS strains were detected in either group. Overall, *S. aureus* demonstrated a higher prevalence in the ears of healthy dogs compared to those with otitis, while *S. schleiferi* was more frequently isolated from otitic cases. The resistance profile revealed increased resistance to beta-lactams in *S. aureus* and enhanced resistance to lincosamides in *S. schleiferi.*

The results obtained in this study highlight the need for continuous monitoring of antimicrobial resistance profiles, both in clinical infections and among healthy dog populations. The presence of resistant strains in both groups suggests a potential reservoir of resistance genes that warrants attention in routine veterinary practice.

### 3.1. Strengths

This study provides a comprehensive analysis of the prevalence and antimicrobial resistance profiles of *S. aureus* and *S. schleiferi* in dogs with otitis externa as well as in healthy dogs. Additionally, the study includes a detailed assessment of MDR strains, highlighting their proportion and contributing to the understanding of epidemiological risks associated with bacterial resistance in the veterinary environment.

### 3.2. Limitations

This study has several limitations that should be considered when interpreting the findings. First, molecular typing and detection of resistance genes were not performed, which restricted the mechanistic interpretation of the phenotypic resistance patterns. Subspecies differentiation of Staphylococcus was also not conducted, potentially obscuring subspecies-specific antimicrobial resistance tendencies.

The geographical and temporal scope of the sample set was relatively narrow, limiting broader extrapolation of prevalence and AMR trends. No statistical tests of significance were applied, and clinical correlation, such as treatment history or otoscopic severity, could not be assessed due to lack of clinical metadata.

Furthermore, the investigation focused solely on *S. aureus* and *S. schleiferi*. Comprehensive characterization of the broader otic microbiome and potential co-infection patterns was beyond the scope of the present study, which may limit contextual interpretation of the bacterial dynamics involved.

## 4. Materials and Methods

### 4.1. Study Design

The research involved collecting specimens from canines affected by otitis externa, together with clinically healthy dogs brought in for routine veterinary care such as vaccination, deworming, and sterilization, at multiple clinics across western Romania. The study was carried out from 2022 to 2025, with samples collected immediately after the animals arrived at the clinic.

### 4.2. Sampling

Ear exudate samples were collected from both ears of 973 dogs, of which 503 showed clinical signs consistent with otitis externa, including pruritus, head shaking, erythema, localized pain, and discharge, while 470 were clinically healthy. Sterile cotton swabs were used to obtain samples, which were then transferred into Amies transport medium and kept at 4 °C until laboratory processing, within a 24-h timeframe. Although bilateral sampling was performed, a single isolate per animal was selected for analysis. This approach was taken to avoid redundancy in cases where *S. aureus* or *S. schleiferi* isolates from both ears of the same dog shared identical resistance profiles. Additionally, there were dogs from which the targeted species was isolated from just one ear, while the contralateral sample either yielded a different bacterium or no growth at all.

### 4.3. Bacterial Isolation and Identification

Samples were inoculated onto Columbia blood agar and Mannitol Salt Agar plates, then incubated aerobically at 37 °C for 24–48 h to promote growth of *Staphylococcus* spp. Presumptive colonies were identified according to their typical morphological features observed on the culture media (e.g., golden-yellow colonies for *S. aureus*, and white to cream colonies for *S. schleiferi*) and hemolysis patterns.

Presumptive colonies were transferred onto fresh Columbia blood agar plates to ensure isolation of pure cultures before species identification.

Prior to performing antimicrobial susceptibility assessments, presumptive bacterial isolates belonging to *Staphylococcus* spp. and were taxonomically characterized using matrix-assisted laser desorption/ionization time-of-flight mass spectrometry (MALDI-TOF MS), employing the Microflex™ system (Bruker Daltonik, Bremen, Germany). Protein extraction from bacterial colonies was conducted using a standardized ethanol/formic acid-based preparation method. A 1 μL aliquot of the resulting protein solution was deposited onto a MALDI target plate, followed by the application of 1 μL of matrix solution containing α-cyano-4-hydroxycinnamic acid (10 mg/mL), dissolved in a solvent mixture of 50% acetonitrile with 2.5% trifluoroacetic acid. Mass spectra were generated through analysis with the Microflex™ mass spectrometer provided by (Bruker Daltonik) and interpreted through the MALDI BioTyper™ 3.0 platform (Bruker Daltonik GmbH, Bremen, Germany). Identification was achieved by matching the acquired spectra against the manufacturer-provided database, in accordance with Bruker’s interpretative criteria: a score ≥ 2.0 denoted species-level identification, whereas values ranging from 1.7 to 1.99 indicated classification at the genus level [48].

To ensure accurate species identification, quality control was carried out by employing the reference strain *S. aureus* ATCC 25923, alongside the Bacterial Test Standard (BTS), in strict accordance with the Bruker protocol. This step guaranteed the reliability and accuracy of the MALDI-TOF MS identification process. The Biotyper database used for identification included manufacturer-approved protein spectra for *S. aureus* and *S. schleiferi.*

### 4.4. Antimicrobial Susceptibility Testing (AST)

Antimicrobial susceptibility evaluation was conducted using the automated VITEK^®^ 2 Compact system (bioMérieux, Marcy-l’Étoile, France), in accordance with the manufacturer’s guidelines. Initially, pure bacterial cultures were obtained, and microbial suspensions were formulated using sterile saline medium, adjusting turbidity to align with the 0.5 McFarland standard. To determine resistance profiles, specific cards tailored to the bacterial classification were employed, Vitek AST-GP 79 (bioMérieux) for Gram-positive strains [48].

Antimicrobial susceptibility testing was carried out using a panel of antimicrobial agents representing diverse antimicrobial classes, as follows: β-lactams (penicillin, oxacillin); aminoglycosides (gentamicin); tetracyclines (tetracycline); fluoroquinolones (ciprofloxacin, moxifloxacin); macrolides (erythromycin); lincosamides (clindamycin); oxazolidinones (linezolid); glycopeptides (teicoplanin, vancomycin); fusidanes (fusidic acid); glycylcyclines (tigecycline); and sulfonamides + pyrimidines (trimethoprim-sulfamethoxazole).

The present study evaluated antimicrobial resistance based solely on phenotypic susceptibility profiles. Antimicrobial susceptibility results were interpreted following the Clinical and Laboratory Standards Institute (CLSI) VET01, Fifth Edition (2018) guidelines [48]. The VITEK^®^ 2 Compact system underwent daily quality assurance procedures in accordance with the manufacturer’s instructions, using *S. aureus* ATCC 29213 as the control strain. The VITEK^®^ 2 Compact is a semi-automated MIC-based system, and results were interpreted according to CLSI VET01 (5th edition, 2018) [48].

Both reference strains were maintained and tested under the same laboratory conditions as field isolates, ensuring reliability and reproducibility of results.

### 4.5. Ethical Approval

This study received approval from the Bioethics Commission of the University of Life Sciences “King Mihai I” in Timișoara (Approval No. 598, dated 10 October 2025). No live animals were subjected to experimental procedures, and all activities fully complied with ethical standards governing animal research.

## 5. Conclusions

This study provides new insights into the prevalence and antimicrobial resistance profiles of *S. aureus* and *S. schleiferi* isolated from dogs with otitis externa, as well as from clinically healthy dogs in western Romania. Notably, *S. aureus* was more frequently isolated from the ears of healthy dogs than from those with clinical signs of otitis, whereas *S. schleiferi* was more commonly associated with otitic samples.

Among the antimicrobial agents tested, the highest resistance rates in *S. aureus* were observed for β-lactams, particularly penicillin, while *S. schleiferi* isolates exhibited the greatest resistance to clindamycin, a lincosamide. MDR strains were more prevalent in isolates from dogs with otitis externa than in those from healthy dogs, emphasizing the clinical significance of resistant staphylococcal populations in infection contexts. Importantly, MRSA strains were detected exclusively in healthy dogs, and no MRSS strains were identified.

The presence of antimicrobial-resistant strains, including MDR and methicillin-resistant phenotypes, in both diseased and healthy canine populations highlights the potential for asymptomatic carriage and environmental dissemination of resistance determinants. These findings underscore the importance of implementing routine antimicrobial susceptibility testing in veterinary practice, along with ongoing surveillance efforts, to support evidence-based therapeutic decisions and minimize the risk of treatment failure. Furthermore, the detection of resistant strains in clinically healthy dogs calls attention to their role as potential reservoirs of antimicrobial resistance in the community.

## Figures and Tables

**Table 1:** Antimicrobial resistance profiles of *S. aureus* and *S. schleiferi* isolates from dogs with otitis externa and from the ears of healthy dogs.

Tested Antimicrobial Agent	*S. aureus* Otitis (*n* = 20)		*S. aureus* Healthy (*n* = 94)		*S. schleiferi* Otitis (*n* = 38)		*S. schleiferi* Healthy (*n* = 18)	
	S (%)	R (%)	S (%)	R (%)	S (%)	R (%)	S (%)	R (%)
Penicillin	35	65	45.7	54. 3	71.05	28.9	83.3	16.7
Oxacillin	100	0	95.7	4.3	100	0	100	0
Gentamicin	90	10	100	0	89.5	10.5	100	0
Tetracycline	65	35	67	33	78.95	21.05	94.4	5.6
Ciprofloxacin	100	0	85.1	14.9	100	0	100	0
Moxifloxacin	100	0	100	0	100	0	100	0
Erythromycin	95	5	100	0	89.5	10.5	100	0
Clindamycin	65	35	66	34	55.3	44.7	55.6	44.4
Linezolid	100	0	100	0	100	0	100	0
Teicoplanin	100	0	100	0	100	0	100	0
Vancomycin	100	0	97.9	2.1	100	0	100	0
Fusidic acid	100	0	97.9	2.1	100	0	100	0
Tigecycline	100	0	100	0	100	0	100	0
Trimethoprim + sulfamethoxazole	85	15	91.5	8.5	100	0	100	0

**Table 2:** Distribution of antimicrobial resistance by antimicrobial class in *S. aureus* and *S. schleiferi* strains isolated from dogs with otitis externa and from the ears of healthy dogs.

Antimicrobial Class	*S. aureus* Otitis (*n* = 20)	*S. aureus* Healthy (*n* = 94)	*S. schleiferi* Otitis (*n* = 38)	*S. schleiferi* Healthy (*n* = 18)
	S/R %	S/R %	S/R %	S/R
Beta-lactams	35/65	45.7/54.3	71.05/28.9	83.3/16.7
Aminoglycosides	90/10	90/10	89.5/10.5	100/0
Tetracyclines	65/35	67/33	78.95/21.05	94.4/5.6
Fluoroquinolones	100/0	85.1/14.9	100/0	100/0
Macrolides	95/5	100/0	89.5/10.5	100/0
Lincosamides	65/35	66/34	55.3/44.7	55.6/44.4
Oxazolidinones	100/0	100/0	100/0	100/0
Glycopeptides	100/0	97.9/2.1	100/0	100/0
Fusidane antibiotics	100/0	97.9/2.1	100/0	100/0
Glycylcyclines	100/0	100/0	100/0	100/0
Sulfonamides + Pyrimidines	85/0	91.5/8.5	100/0	100/0

**Table 3:** Antimicrobial resistance profiles of individual *S. aureus* strains isolated from dogs with otitis externa.

*S. aureus* (*n* = 20)	
Antimicrobial resistance	Number of strains and percentage
PEN	8 (40%)
CLI	2 (10%)
TET	2 10%)
PEN + CLI	2 (10%)
PEN + TET	1 (5%)
TET + CLI + SXT	2 (10%)
PEN + TET + ERY	1 (5%)
PEN + GEN + SXT	1 (5%)
GEN + TET + CLI	1 (5%)

Legend: PEN—penicillin; CLI—clindamycin; TET—tetracycline; ERY—erythromycin; GEN—gentamicin; SXT—trimethoprim + sulfamethoxazole.

**Table 4:** Antimicrobial resistance profiles of individual *S. aureus* strains isolated from the ears of healthy dogs.

*S. aureus* (*n* = 94)	
Antimicrobial resistance	Number of strains and percentage
PEN	34 (36.2%)
TET	13 (13.8%)
CLI	12 (12.8%)
SXT	4 (4.3%)
CLI + CIP	11 (11.7%)
PEN + TET	5 (5.3%)
PEN + TET + CLI	4 (4.3%)
PEN + TET + SXT	4 (4.3%)
TET + CIP + CLI	3 (3.2%)
PEN + OXA + VAN + FUS	1 (1.1%)
PEN + OXA + TET + CLI + FUS	1 (1.1%)
PEN + OXA + VAN + TET	1 (1.1%)
PEN + OXA + TET + CLI	1 (1.1%)

Legend: PEN—penicillin; TET—tetracycline; CLI—clindamycin; SXT—trimethoprim + sulfamethoxazole; CIP—ciprofloxacin; OXA—oxacillin; VAN—vancomycin; FUS—fusidic acid.

**Table 5:** Antimicrobial resistance profiles of individual *S. schleiferi* strains isolated from dogs with otitis externa.

*S. schleiferi* (*n* = 38)	
Antimicrobial resistance	Number of strains and percentage
Susceptible to all tested antibiotics	5 (13.2%)
PEN	5 (13.2%)
CLI	12 (31.6%)
TET	4 (10.5%)
GEN	3 (7.9%)
ERY	2 (5.3%)
PEN + TET	2 (5.3%)
GEN + CLI	1 (2.6%)
PEN + TET + CLI	2 (5.3%)
PEN + ERY + CLI	2 (5.3%)

Legend: PEN—penicillin; CLI—clindamycin; TET—tetracycline; GEN—gentamicin; ERY—erythromycin.

**Table 6:** Antimicrobial resistance profiles of individual *S. schleiferi* strains isolated from the ears of healthy dogs.

*S. schleiferi* (*n* = 18)	
Antimicrobial resistance	Number of strains and percentage
Susceptible to all tested antibiotics	8 (44.4%)
CLI	7 (38.9%)
PEN	2 (11.1%)
PEN + TET + CLI	1 (5.6%)

Legend: PEN—penicillin; TET—tetracycline; CLI—clindamycin.

## Data Availability

The original contributions presented in this study are included in the article. Further inquiries can be directed to the corresponding author.

## Abbreviations

The following abbreviations are used in this manuscript:

AMR	Antimicrobial resistance
MDR	Multidrug-resistant
MRSA	Methicillin-resistant *Staphylococcus aureus*
*S. schleiferi*	*Staphylococcus schleiferi*
MRSS	Methicillin-resistant *Staphylococcus schleiferi*
MALDI-TOF MS	Matrix-assisted laser desorption/ionization time-of-flight mass spectrometry
AST	Antimicrobial Susceptibility Testing
CLSI	Clinical and Laboratory Standards Institute
